# On Giving Rest to the Digestive Organs by the Use of Peptonised Food

**Published:** 1884-03

**Authors:** W. H. Spencer

**Affiliations:** Senior Physician to the Bristol Royal Infirmary


### ON GIVING REST TO THE DIGESTIVE ORGANS BY THE USE OF PEPTONISED FOOD.

BY

W. H. SPENCER, M.A., M.D. Cantab.,

*Senior Physician to the Bristol Royal Infirmary.*

---

EVERYONE knows and understands the great need for giving rest to organs in the carrying out of a rational line of treatment—especially in regard to organs which are inflamed. We often want in our practice to give entire repose to organs; yet we have to confess our inability, for want of knowledge and means, to fulfil the indication; again, having the means at hand, we hesitate lest the very inactivity we *could* bring about should imperil the well-being of other organs or even of the whole being. But our ignorance and inability of to-day give place to-morrow to knowledge and ability; hesitation is changed to confidence and safe action. We must always have a great deal more of physiological knowledge than we can make use of. Yet many workers are digging at the physiological quarry, and continually material is being carried away to be used with effect in the building up of our ever-perfecting house of Practical Medicine. And a good deal of shaping and fitting is needed, and much patient trial, before a method or a means can be safely considered fit for the storehouse of practical precepts—to be acted upon with certainty, by all, for the common good.

Now, it is not so very long ago that there was got out of the physiological quarry some material which, now shaped and tried, is at the disposal of everyone to secure absolute physiological rest for the stomach in many conditions where such rest may be more or less urgently needed. The preparation and use of peptonised food is one of the most recent and acceptable of the many practical applications of physiological science to practical medicine. In this paper I propose to speak of the treatment by peptonised food in general—its foundations in physiology—and this as a prelude to the examples of its use in a particular form of disease—gastric ulcer, to wit—which are given in another part of this book.

But first let me say a few words about this physiological rest that we so often desire to compass as preliminary to our treatment, or as the chief part of it. The term itself, physiological rest, is perhaps not a good one, but I mean this by it—the relief of an organ from some or all of the work incident to its special functions. Organs have work to do beyond that which is necessary to their own life, and it is from this additional and, for each organ, special work we often need to spare organs. As yet, it must be confessed, we are very far from knowing how such relief and rest *could* be given to most organs of the body. In respect of the brain or spinal cord, for instance, with such means as we have, unless we extinguish altogether their proper functions, we are confronted with incidents and consequences which make us hesitate to use our means except when urgency and hopelessness demand our action.

Disease may sometimes secure rest from special functional activity whilst not extinguishing the life of the organ or part. Special function is in abeyance in the lower lobe of a lung when it is the seat of croupous pneumonia; air is excluded, aeration is impossible, all the activities pertaining to the special function of the lung are laid to rest. Now, in certain forms of phthisis the lung has not only to bear the burden of its own diseased state, but also the burden of its special function, and this when already lamed by disease. So that, the having this rest may be in the pneumonic lung a help, and the want of it may be in the phthisical lung a hindrance to the proper recovery from their respective diseased conditions. We often desire in our treatment to give rest to organs by restraining mechanical movement. Thus, we may strap the chest in pleurisy; or restrain peristaltic action in the intestine by means of opium. But our task is not so easily made good in regard to most organs.

The stomach is a very difficult organ to deal with. To give the stomach the complete rest we often need to give it we must arrest motion *and* special function. In the presence of structural disease it will not do to use half-measures. There is nothing for it, in order to give the stomach relief from all its work, but to withhold from it all ordinary proteid food, all stimulus of whatever kind.

Let me recall some of the knowledge we have as to the stomach and its work. As to the movements of the stomach: these do not seem to depend on mere fulness of the organ, or amount of food in it; they are slight and feeble at first after a meal and when the stomach is fullest; they grow in strength as digestion goes on; they keep pace with the acidity of the contents of the stomach—the more acid the more movement, the less acid the less movement. When quite empty the stomach shows none of its peculiar muscular action, and it is then contracted. We know little of the nervous mechanism of the gastric movements. It is, however, significant that after section of the vagi (the only nerves that seem to be concerned in the movements of the stomach), and irritation of the peripheral ends, movements are set up *only* if the stomach is full; there is no response when the organ is empty.

As to the special function of the stomach, its secretion of gastric juice: We know that slight mechanical stimulation causes a slight pouring out of the secretion; that the greatest amount of effect in this direction is caused by the presence in the stomach of a dilute alkali—even the presence of a small quantity of saliva causes a flow of gastric juice. When the stomach is empty the mucus membrane is pale and grey; the surface of the empty stomach is barely moist, and yields no decided acid reaction (Leube). The influence of nerve-action upon the secretion of gastric juice seems to be small.

From all we know, it would seem as if the amount of muscular action in the stomach depended more upon the quality and state of the contents of the organ than upon mere mechanical stimulus. And as regards secretion of gastric juice, it would seem as if this, too, depended upon the quality and state—especially the chemical state—of the contents of the stomach.

I think we may say this with certainty—that we *can* arrest motion *and* secretion in the stomach, and so give physiological rest to the organ. But we can only certainly and completely arrest motion *and* secretion by entire withholding of food.

Yet, after the reflection which all the facts of the case invite, it seems to me that some further considerations are suggested. The actions of the stomach (both muscular and secretory) seeming to depend upon qualitative properties in the food—whatever they may be,—it is as if the actions went hand in hand with the amount of work required to be done. And we get this suggestion, which may be in the nature of a principle, that the more the ingesta approach the qualitative state of the completely digested food—in so far as stomach digestion is concerned—the less will the activities of the stomach, both muscular and secretory, be called forth. Certain it is that the clinical results we gain by acting as if this suggestion were a well-ascertained principle go far to confirm its reasonableness and its foundation in fact. Wherefore, I think we may go further and say: that we may rest the stomach more or less by feeding it with ingesta already so altered in state and quality as to be more or less in the state and quality of matters which have undergone complete digestion.

In some organs function goes on well enough for the general wants of the body, even though a part of the organ be inactive from disease. But, for the stomach, abeyance of special function means a cutting off of the source of nourishment and life from all other organs, and even the whole organism. If we cut off all supply of nutrient material through the stomach, we must, to support life, find some means not only to convey nutrient material into the circulation, but to convey it in a digested form—and this means in something more than a soluble form.

Everyone knows that the work done by the stomach is upon the proteid elements of food; that under the joint influence of heat, acid and ferment the proteid food-stuffs dissolve and break up, and become changed into highly diffusible peptones. The work is not, however, quite so simple as it may appear to be. The process of gastric digestion is a complex process, and not yet so unravelled as to enable us to dispense with theory.

Speaking broadly, the process is essentially a chemical process—of that kind called *hydration*, or hydrolytic decomposition—whereby the large, complex and unstable molecules of protein split up into definite and more stable constituent substances; whilst at the same time a change in physical state occurs, the insoluble colloid being changed into soluble and highly diffusible crystalloids. This change is effected through the agency of the ferment, pepsin, in the presence of water and acid. And a salient point in regard to these changes is this: that in the more strictly chemical metamorphoses during digestion molecules are so re-arranged as to provide for re-conversion, after absorption, into the proteid form again, and thus to aid in those synthetic changes upon which the building-up of tissues depends. The change from proteid to peptone, in the stomach, is effected in stages, and with the appearance of intermediate products. A proteid, undergoing stomach, or peptic, digestion, seems to split up chiefly into two groups—so called anti-albumose and hemi-albumose—from each of which two somewhat differing peptones are formed. One of these peptones—anti-peptone—is the final and completed product; it is complete in those properties which fit it for immediate introduction into the circulation, and accordingly, in the stomach, it is at once absorbed. The other peptone—hemi-peptone—may be said to be incomplete; it is capable of being still further split up and changed before absorption. But not in the stomach or by peptic digestion. This further change takes place in the small intestine, and through the agency of the ferment of the pancreatic juice, trypsin, in the presence of water and alkali.

As is well known, the pancreatic juice is capable of converting proteids into peptones; and the mode of conversion is on the same lines as in the case of the gastric juice, yet differing in important ways. When trypsin acts, in proper conditions, upon a proteid, two groups of intermediate products are formed—anti-albumose and hemi-albumose. Comparing these two with the corresponding products of peptic digestion, we find that there are certain differences between the tryptic and peptic products: those formed in the stomach are akin to syntonin or acid-albumin; those formed in the intestine are akin to alkali-albumin. Further, whilst pepsin has no power to split up or otherwise alter the properties of either of its peptones, trypsin has the power to split up and alter one of its peptones, the hemi-peptone, to wit. Pancreatic juice can do, in fact, all that gastric juice can do in the way of peptonising proteids, and something more—it can split up hemi-peptone into leucin and tyrosin and other less important things. Now leucin is a fatty body (amido-caproic acid), and tyrosin belongs to the aromatic group, and is akin to benzoic acid; they are crystalline nitrogenous bodies, and their capacities in regard to metabolism are great. Besides the digestion of proteids, pancreatic juice can effect the digestion of fats (aided by the bile) and of starch.

The question next arises—Can we imitate and bring about out of the body the processes of digestion, with the results just now set forth? If we can, the tryptic or pancreatic process of digestion is clearly the best to imitate. And there is no longer any doubt that we can in all essential points bring about out of the body to raw material the same changes that tryptic or pancreatic digestion brings about in the bowel to our food. Long ago, by means of pepsin (prepared artificially from the actively-digesting stomach of the pig) and acid and heat, the process of gastric digestion was fairly imitated out of the body. At the present day, by means of pancreatic extract and alkali and heat, the more complete process of intestinal digestion is imitated almost exactly and easily out of the body, and now daily in our hospitals, and even in private houses. And in this way a great variety of pleasant foods are prepared, whose use, in a ready-digested form, is attended with much success.

As to the means used in artificial digestion—peptonisation, as it is called—first, we must have the special ferment. It is found that by treating the pancreas of the pig with dilute spirit a very active extract of the pancreatic juice can be obtained. An active and efficient extract of fairly constant strength is now made on a large scale in commerce, the extract known as Benger's Liquor Pancreaticus being, perhaps, the best. The natural ferment being thus at hand, for the rest it is a mere matter of being able to secure the proper physical conditions—temperature, and alkaline state, and time—in order to digest artificially almost any of the chief food-stuffs we please.*

Now, finally, in order to secure complete rest to the stomach, we need to introduce into the circulation the food we can thus artificially digest through some channel other than the vessels or membranes of the stomach and small intestine. It is well known how little we could do in the way of supporting life, for more than a day or two, by the use of what I must now call the old-fashioned nutrient injection. And the reason is clear: we could

* For details as to the preparation of peptonised milk, beef-tea, gruel, and other farinaceous foods, consult the article on Peptonised Food, by Dr. William Roberts, in Quain's *Dictionary of Medicine*, or Dr. Roberts' *Lumleian Lectures*.

not reduce the food before injection into the rectum to a soluble and peptonic form. With the means now at our disposal we can, out of the body, reduce proteids to the soluble and peptonic form; and we can at the same time, and by the same agency, convert starch into dextrin and sugar, and we can peptonise milk. We can prepare an enema which is, in its likeness to digested chyle, nutrient indeed. We are, therefore, limited only by the capacity of the rectum to absorb such food, in our efforts to support life for many weeks whilst we give the whole alimentary canal absolute rest from all digestive work. Now, it has been established experimentally that peptones and sugar have been completely absorbed by the large intestine after injection *per anum*. We need, I think, no other scientific proof than this of the certainty of absorption after rectal injection, of soluble and peptonised food.

